# NeoFuse: predicting fusion neoantigens from RNA sequencing data

**DOI:** 10.1093/bioinformatics/btz879

**Published:** 2019-11-22

**Authors:** Georgios Fotakis, Dietmar Rieder, Marlene Haider, Zlatko Trajanoski, Francesca Finotello

**Affiliations:** Biocenter, Institute of Bioinformatics, Medical University of Innsbruck, Innsbruck 6020, Austria

## Abstract

**Summary:**

Gene fusions can generate immunogenic neoantigens that mediate anticancer immune responses. However, their computational prediction from RNA sequencing (RNA-seq) data requires deep bioinformatics expertise to assembly a computational workflow covering the prediction of: fusion transcripts, their translated proteins and peptides, Human Leukocyte Antigen (HLA) types, and peptide-HLA binding affinity. Here, we present NeoFuse, a computational pipeline for the prediction of fusion neoantigens from tumor RNA-seq data. NeoFuse can be applied to cancer patients’ RNA-seq data to identify fusion neoantigens that might expand the repertoire of suitable targets for immunotherapy.

**Availability and implementation:**

NeoFuse source code and documentation are available under GPLv3 license at https://icbi.i-med.ac.at/NeoFuse/.

**Supplementary information:**

Supplementary data are available at *Bioinformatics* online.

## 1 Introduction

Neoantigens are tumor-specific peptides arising from the expression of mutated genes in cancer cells. Class-I neoantigens, recognized as ‘non-self’ by CD8^+^ T cells, can elicit strong anticancer immune responses. Besides being major determinants of response to immune checkpoint blockers, neoantigens are at the basis of other cancer immunotherapies like personalized cancer vaccines and adoptive T cell therapy (Lee *et al.*, 2018).

To date, most efforts have been directed at identifying neoantigens generated from missense somatic mutations (Finotello *et al.*, 2019). However, tumor-specific gene fusions, splicing isoforms, and expressed human endogenous retroviruses can also be a source of neoantigens (Smith *et al.*, 2019). A recent study in patients with head and neck cancer demonstrated that gene fusions generate immunogenic neoantigens that can mediate the response to immune checkpoint blockers in tumors with low mutational burden (Yang *et al.*, 2019).

Computational strategies for the identification of fusion neoantigens from RNA sequencing (RNA-seq) data have been proposed recently (Rathe *et al.*, 2019; Richman *et al.*, 2019; Zhang *et al.*, 2017). However, all of them build upon pre-analysis with third-party tools to first predict fusion transcripts from tumor RNA-seq and, thus, deep bioinformatic expertise for the assembly of a full computational workflow.

Here, we present NeoFuse, a user-friendly pipeline for the prediction of fusion neoantigens from tumor RNA-seq data. NeoFuse is available as Singularity (https://sylabs.io) and Docker (https://www.docker.com) images to simplify installation and analysis.

## 2 The NeoFuse pipeline

**Fig. 1.**
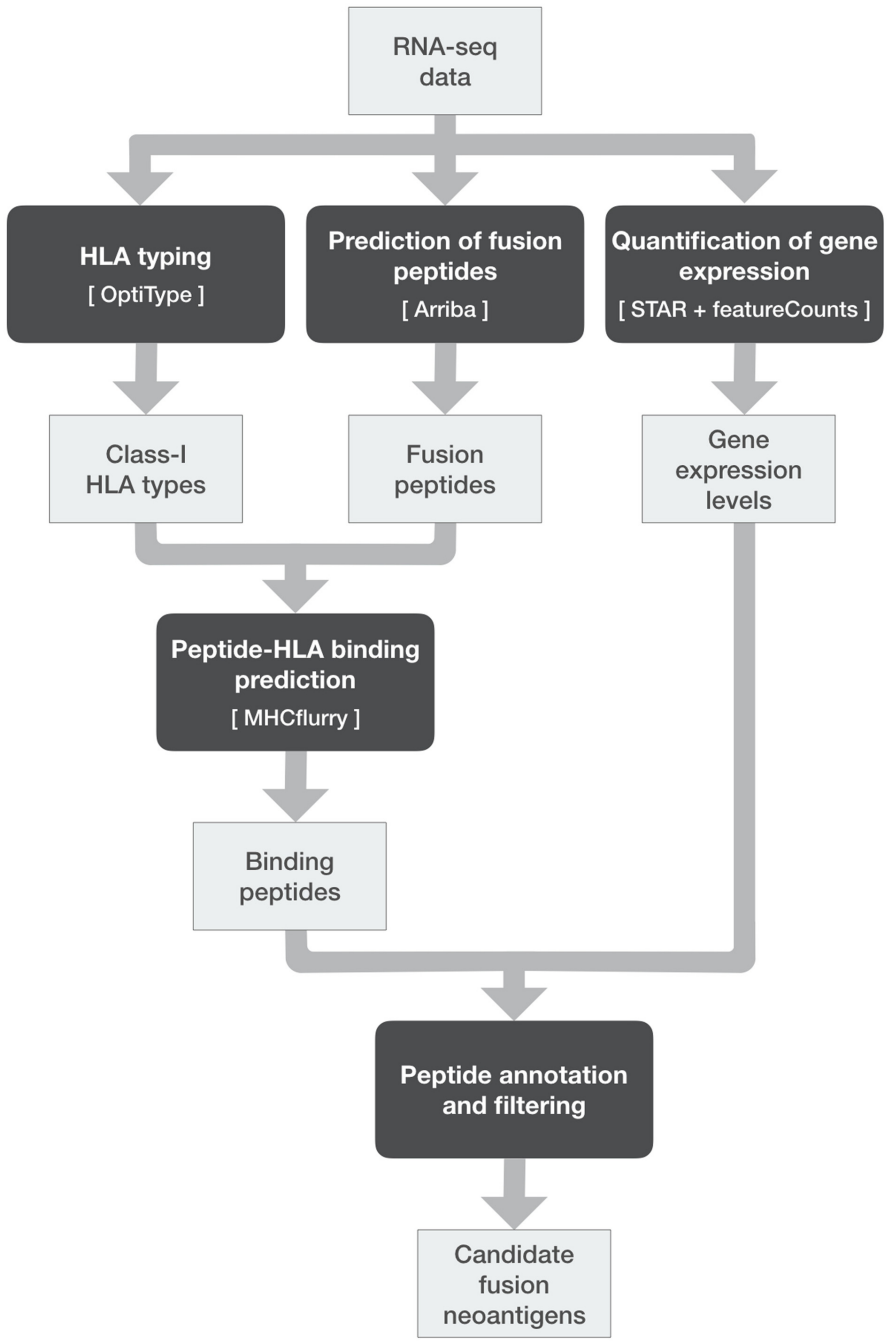
Schematization of the NeoFuse pipeline: computational modules represented as dark-grey boxes (with tool names in square brackets) and output files as light-grey boxes

NeoFuse takes single-sample FASTQ files of RNA-seq reads as input and predicts putative fusion neoantigens through five analytical modules based on state-of-the-art computational tools (Fig. 1). Both single- and paired-end data can be used, but we advise using the latter to increase sensitivity and accuracy of fusion detection. The first module performs class-I Human Leukocyte Antigen (HLA) typing at 4-digit resolution using OptiType (Szolek *et al.*, 2014), which is one of the best performing methods for this task (Finotello *et al.*, 2019). The second module predicts fusion peptides using Arriba (https://github.com/suhrig/arriba), considering both fusion junctions and 3’ out-of-frame sequences. We chose Arriba because it outperformed competitor prediction methods in the DREAM Somatic Mutation Calling–RNA Challenge (https://www.synapse.org/SMC_RNA). Moreover, it computes a confidence score reflecting the likelihood that a fusion is caused by a tumor-specific genomic rearrangement and is not due to technical artifacts. The third module uses MHCflurry (O’Donnell *et al.*, 2018) to predict binding affinity of fusion peptides to HLA types, quantified as half maximal inhibitory concentration (IC50) and percentile rank. The fourth module leverages STAR (Dobin *et al.*, 2013) and featureCounts (Liao *et al.*, 2014) to quantify gene expression levels as transcripts per million. Finally, the fifth module selects a reduced set of peptides representing putative fusion neoantigens by considering their binding affinity and confidence score. Moreover, it annotates each neoantigen with IC50, percentile rank, confidence score, binding HLA type, expression of fusion and HLA genes and information about the presence of a premature stop codon that might cause nonsense-mediated decay of the fusion transcript.

NeoFuse is available as ready-to-use Singularity and Docker images containing all the necessary software and dependencies. This allows running the pipeline in an isolated environment, preventing conflicts with other programs in the hosting environment. Local installation of the images is performed automatically by the NeoFuse bash script depending on the user’s choice (‘–docker’ or ‘–singularity’ option). Although not distributed as part of NeoFuse, netMHCpan (Jurtz *et al.*, 2017) can be used for peptide-HLA binding prediction instead of MHCflurry, provided that a local installation is available (see online documentation).

## 3 Applications

To assess the performance of the gene fusion prediction module, we tested Arriba and other state-of-the-art tools on two benchmark RNA-seq datasets (Supplementary Fig. S1). When used with the ‘-c M’ parameter setting to select fusions with medium and high confidence scores, Arriba together with STAR-Fusion (Haas *et al.*, 2017) resulted the best performer in terms of validated fusions identified, while also limiting the total number of called fusions. More aggressive or conservative solutions could be obtained using the ‘-c L’ or ‘-c H’ options, respectively. The analysis of each dataset with Arriba took, on average, 6 min on a high performance computing node (HP XL230a in Apollo 6000) utilizing 10 cores (Intel E5-2699A v4, 2.4 GHz) per sample.

As a test case, we analyzed eight RNA-seq datasets from the MCF7 breast cancer cell line (Supplementary Table S1), selecting fusions with medium and high Arriba confidence score and peptides with an IC50 lower than 500 nM (‘-t 500’ option). On average, we identified 144 putative neoantigens from 40 gene fusions, with 83.96% of gene fusions characterized by out-of-frame sequences. The latter result suggests that gene fusions can be a source of neoantigens whose sequences are extremely different from that of self-peptides. Fusions shared across all datasets included gene pairs previously validated experimentally (BCAS4-BCAS3) or identified with computational methods (ABCA5-PPP4R1L, DEPDC1B-ELOVL7) (Picco *et al.*, 2019). OptiType predicted the correct HLA genotypes for the SRR1035698 dataset, but called homozygous HLA-B alleles for the datasets with a low expression (Supplementary Table S2) and, thus, read coverage of this gene (Supplementary Fig. S2).

## 4 Conclusions

NeoFuse is a novel computational pipeline to predict fusion neoantigens from tumor RNA-seq data. It is based on state-of-the-art computational tools and is available as ready-to-use Singularity and Docker images to ease installation and usage, requiring limited bioinformatic expertise. NeoFuse can be easily applied to RNA-seq data from patients with different cancer types. Thus, it can be used to identify fusion neoantigens that can broaden the repertoire of candidates for therapeutic cancer vaccination and T cell-based therapy and might ultimately extend the clinical benefit of immunotherapy to patients with low tumor mutational burden. In the near future, we plan to extend NeoFuse to the prediction of class-II fusion neoantigens recognized by CD4^+^ T cells.

## Funding

This work was supported by the Austrian Cancer Aid/Tyrol [project n. 17003 to F.F.], by the Austrian Science Fund (FWF) [project n. T 974-B30 to F.F.] and by the European Research Council [advanced grant agreement n. 786295 to Z.T.].

*Conflict of Interest*: none declared. 

## Supplementary Material

btz879_Supplementary_Data

## References

[btz879-B1] Dobin A. et al (2013) Star: ultrafast universal RNA-seq aligner. Bioinformatics, 29, 15–21. 23104886 10.1093/bioinformatics/bts635 PMC3530905

[btz879-B2] Finotello F. et al (2019) Next- generation computational tools for interrogating cancer immunity. Nat. Rev. Genet., 20, 724. 31515541 10.1038/s41576-019-0166-7

[btz879-B3] Haas B. et al (2017) STAR-fusion: fast and accurate fusion transcript detection from RNA-seq. BioRxiv, 120295. doi: 10.1101/120295.

[btz879-B114] Jurtz V. et al (2017) NetMHCpan-4.0: improved peptide MHC class I interaction predictions integrating eluted ligand and peptide binding affinity data. J Immunol., 199, 3360–3368. 28978689 10.4049/jimmunol.1700893 PMC5679736

[btz879-B4] Lee C.-H. et al (2018) Update on tumor neoantigens and their utility: why it is good to be different. Trends Immunol., 39, 536–548. 29751996 10.1016/j.it.2018.04.005 PMC7954132

[btz879-B5] Liao Y. et al (2014) Featurecounts: an efficient general purpose program for assigning sequence reads to genomic features. Bioinformatics, 30, 923–930. 24227677 10.1093/bioinformatics/btt656

[btz879-B6] O’Donnell T.J. et al (2018) Mhcflurry: open-source class I MHC binding affinity prediction. Cell Syst., 7, 129–132. 29960884 10.1016/j.cels.2018.05.014

[btz879-B7] Picco G. et al (2019) Functional linkage of gene fusions to cancer cell fitness assessed by pharmacological and CRISPR-Cas9 screening. Nat. Commun., 10, 2198. 31097696 10.1038/s41467-019-09940-1 PMC6522557

[btz879-B8] Rathe S.K. et al (2019) Identification of candidate neoantigens produced by fusion transcripts in human osteosarcomas. Sci. Rep., 9, 358. 30674975 10.1038/s41598-018-36840-z PMC6344567

[btz879-B9] Richman,L.P. et al (2019) Neoantigen dissimilarity to the self-proteome predicts immunogenicity and response to immune checkpoint blockade. Cell Syst., 9, 375–382.e4. 31606370 10.1016/j.cels.2019.08.009 PMC6813910

[btz879-B10] Smith C.C. et al (2019) Alternative tumour-specific antigens. Nat. Rev. Cancer, 1. 31278396 10.1038/s41568-019-0162-4 PMC6874891

[btz879-B11] Szolek A. et al (2014) Optitype: precision HLA typing from next-generation sequencing data. Bioinformatics, 30, 3310–3316. 25143287 10.1093/bioinformatics/btu548 PMC4441069

[btz879-B12] Yang W. et al (2019) Immunogenic neoantigens derived from gene fusions stimulate t cell responses. Nat. Med., 25, 767. 31011208 10.1038/s41591-019-0434-2 PMC6558662

[btz879-B13] Zhang J. et al (2017) Integrate-neo: a pipeline for personalized gene fusion neoantigen discovery. Bioinformatics, 33, 555. 27797777 10.1093/bioinformatics/btw674 PMC5408800

