# Supplementary material for: NeoFuse: predicting fusion neoantigens from RNA sequencing data
Source: Bioinformatics. 2019 Nov 22;36(7):2260–1. doi: 10.1093/bioinformatics/btz879 (PMC7141848; doi:10.1093/bioinformatics/btz879)
Supplement: btz879_Supplementary_Data [file btz879_supplementary_data.pdf]

Supplementary Figure 1 | Performance of computational methods for gene fusion prediction tested on two paired-end RNA-seq datasets (Edgren et al. 2011; Berger et al. 2010) for which a list of PCR-validated fusions was available. Performance was assessed in terms of total gene fusions called ('Tot fusions') and number of validated fusions identified ('True positives'). The tested methods were: STAR-Fusion (Haas et al. 2017), FusionCatcher (Nicorici et al. 2014), EricScript (Benelli et al. 2012), and Arriba (https://github.com/suhrig/arriba). Arriba was run with the '-c L', '-c M', or '-c H' option to select fusions based on their confidence score as explained in the main text.

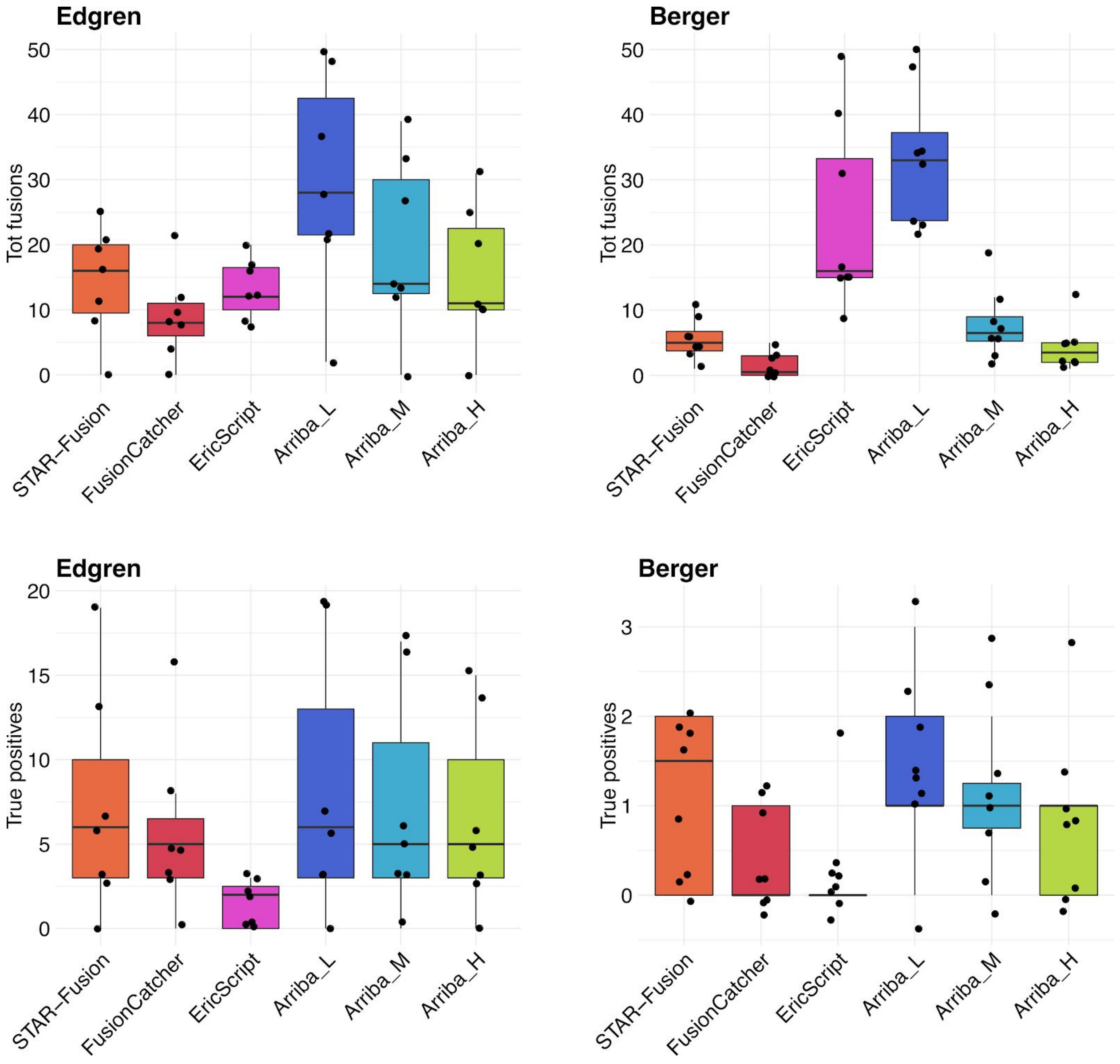

Supplementary Figure 2 | HLA coverage plots generated by OptiType for the SRR1035698 **(a)** and SRR1107834 **(b)** datasets, showing the low HLA-B gene read coverage of the latter.

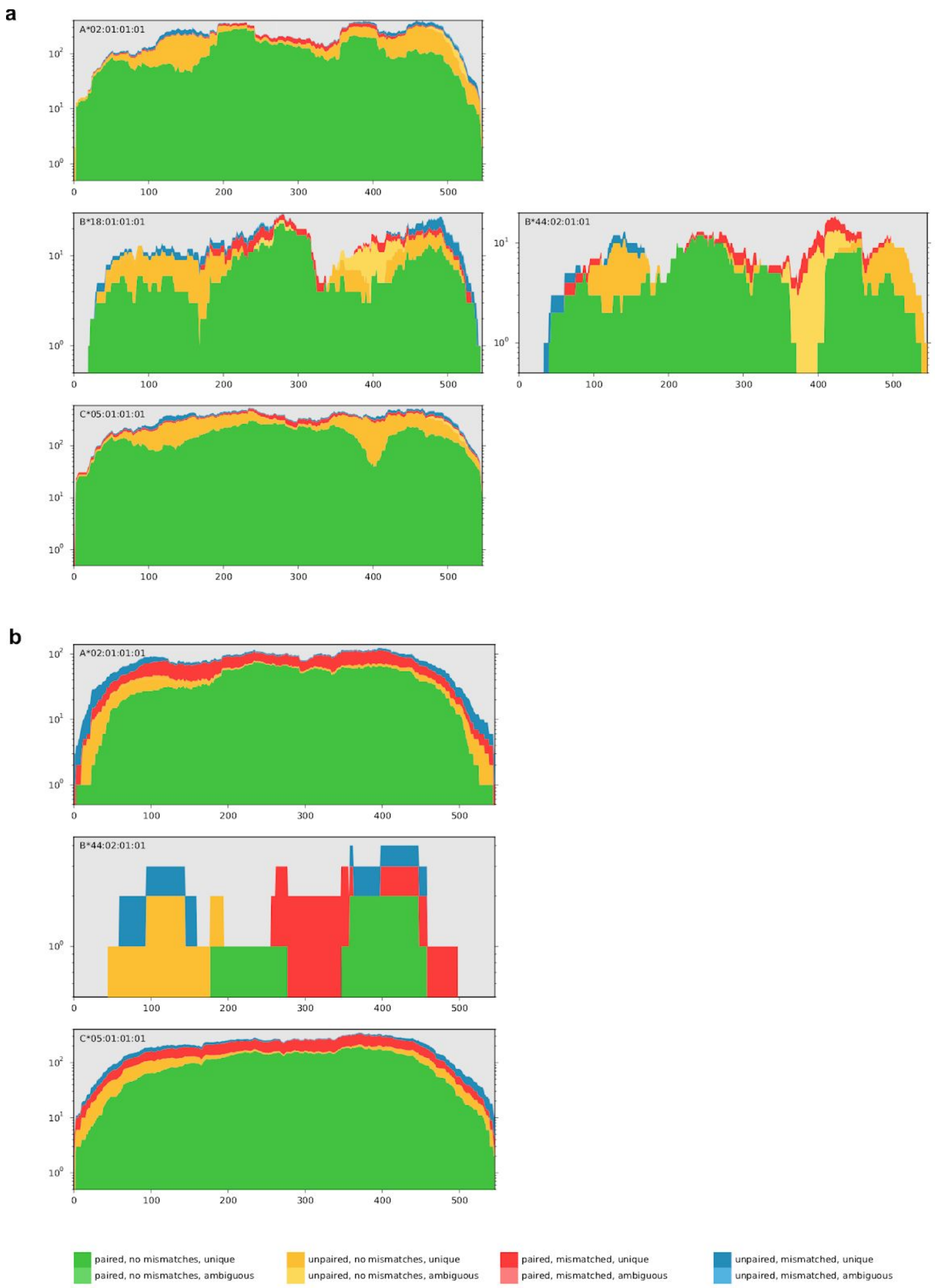

Supplementary Table 1 | Results of NeoFuse analysis of paired-end RNA-seq datasets from the MCF7 breast cancer cell line in terms of: number of fusion neoantigens identified (unique peptides), number of unique neoantigen-generating gene fusions, percentage of out-of-frame gene fusions, and percentage of fusions with premature stop codons. The first column reports the identifiers for data retrieval from the European Genome-phenome Archive (EGA, https://www.ebi.ac.uk/ega/home) or from the Sequence Read Archive (SRA, https://www.ncbi.nlm.nih.gov/sra).

**Dataset identifier**	**Fusion neoantigens**	**Gene fusions**	**Out-of-frame fusions [%]**	**Premature stop codons [%]**
EGAR00001163650	200	58	91.38	65.52
SRR1035698	108	31	83.87	61.29
SRR1107833	155	41	78.05	58.54
SRR1107834	185	40	80.00	55.00
SRR1107835	150	37	81.08	48.65
SRR1313067	97	29	82.76	58.62
SRR2532362	139	45	88.89	57.78
SRR925723	118	35	85.71	62.86

Supplementary Table 2 | Class-I HLA genes results obtained with NeoFuse on paired-end RNA-seq datasets from the MCF7 breast cancer cell line: genotypes and expression levels in transcripts per million (TPM, in brackets). In bold, the MCF7 genotype in agreement with the gold standard defined in (Adams et al. 2005) using high-resolution sequence-based typing.

Dataset identifier	HLA-A	HLA-B	HLA-C
EGAR00001163650	A*02:01 (100.92)	B*18:01 (18.62)	C*05:01 (104.71)
SRR1035698	**A*02:01** (126.09)	**B*18:01-B*44:02** (75.82)	**C*05:01** (176.91)
SRR1107833	A*02:01 (24.55)	B*44:02 (15.83)	C*05:01 (71.05)
SRR1107834	A*02:01 (24.62)	B*44:02 (14.16)	C*05:01 (72.54)
SRR1107835	A*02:01 (23.97)	B*44:02 (17.70)	C*05:01 (71.59)
SRR1313067	A*02:01 (124.04)	B*18:01 (22.84)	C*05:01 (100.91)
SRR2532362	A*02:01 (149.82)	B*18:01 (52.16)	C*05:01 (185.05)
SRR925723	A*02:01 (76.58)	B*44:02 (20.91)	C*05:01 (81.89)

Supplementary References

Adams, Sharon, Fu-Meei Robbins, Deborah Chen, Devika Wagage, Susan L. Holbeck, Herbert C. Morse 3rd, David Stroncek, and Francesco M. Marincola. 2005. “HLA Class I and II Genotype of the NCI-60 Cell Lines.” *Journal of Translational Medicine* 3 (1): 11.

Benelli, Matteo, Chiara Pescucci, Giuseppina Marseglia, Marco Severgnini, Francesca Torricelli, and Alberto Magi. 2012. “Discovering Chimeric Transcripts in Paired-End RNA-Seq Data by Using EricScript.” *Bioinformatics* 28 (24): 3232–39.

Berger, Michael F., Joshua Z. Levin, Krishna Vijayendran, Andrey Sivachenko, Xian Adiconis, Jared Maguire, Laura A. Johnson, et al. 2010. “Integrative Analysis of the Melanoma Transcriptome.” *Genome Research* 20 (4): 413–27.

Edgren, Henrik, Astrid Murumagi, Sara Kangaspeska, Daniel Nicorici, Vesa Hongisto, Kristine Kleivi, Inga H. Rye, et al. 2011. “Identification of Fusion Genes in Breast Cancer by Paired-End RNA-Sequencing.” *Genome Biology* 12 (1): R6.

Haas, Brian J., Alex Dobin, Nicolas Stransky, Bo Li, Xiao Yang, Timothy Tickle, Asma Bankapur, et al. 2017. “STAR-Fusion: Fast and Accurate Fusion Transcript Detection from RNA-Seq.” *bioRxiv*. https://doi.org/10.1101/120295.

Nicorici, Daniel, Mihaela Şatalan, Henrik Edgren, Sara Kangaspeska, Astrid Murumägi, Olli Kallioniemi, Sami Virtanen, and Olavi Kilkku. 2014. “FusionCatcher – a Tool for Finding Somatic Fusion Genes in Paired-End RNA-Sequencing Data.” *bioRxiv*. https://doi.org/10.1101/011650.
